# *In vivo* X-ray elemental imaging of single cell model organisms manipulated by laser-based optical tweezers

**DOI:** 10.1038/srep09049

**Published:** 2015-03-12

**Authors:** Eva Vergucht, Toon Brans, Filip Beunis, Jan Garrevoet, Maarten De Rijcke, Stephen Bauters, David Deruytter, Michiel Vandegehuchte, Ine Van Nieuwenhove, Colin Janssen, Manfred Burghammer, Laszlo Vincze

**Affiliations:** 1 X-ray Microspectroscopy and Imaging Group, Ghent University, Krijgslaan 281 (S12), B-9000 Ghent, Belgium; 2 Department of Electronics and Information Systems, Ghent University, Sint-Pietersnieuwstraat 41, B-9000 Ghent, Belgium; 3 Center for Nano and Biophotonics, Ghent University, Sint-Pietersnieuwstraat 41, B-9000 Ghent, Belgium; 4 Laboratory of Environmental Toxicology and Aquatic Ecology, Ghent University, Jozef Plateaustraat 22, B-9000 Ghent, Belgium; 5 European Synchrotron Radiation Facility, 71 avenue des Martyrs, F-38000 Grenoble, France; 6 Polymer Chemistry and Biomaterials Group, Ghent University, Krijgslaan 281 (S4), B-9000 Ghent, Belgium

## Abstract

We report on a radically new elemental imaging approach for the analysis of biological model organisms and single cells in their natural, *in vivo* state. The methodology combines optical tweezers (OT) technology for non-contact, laser-based sample manipulation with synchrotron radiation confocal X-ray fluorescence (XRF) microimaging for the first time. The main objective of this work is to establish a new method for *in vivo* elemental imaging in a two-dimensional (2D) projection mode in free-standing biological microorganisms or single cells, present in their aqueous environment. Using the model organism *Scrippsiella trochoidea*, a first proof of principle experiment at beamline ID13 of the European Synchrotron Radiation Facility (ESRF) demonstrates the feasibility of the OT XRF methodology, which is applied to study mixture toxicity of Cu-Ni and Cu-Zn as a result of elevated exposure. We expect that the new OT XRF methodology will significantly contribute to the new trend of investigating microorganisms at the cellular level with added *in vivo* capability.

Owing to its high sensitivity, multi-elemental and non-destructive nature, synchrotron radiation (SR) based confocal X-Ray Fluorescence (XRF) imaging offers the unique potential of providing two- and three-dimensional information on the sample composition and elemental distributions with trace level detection limits^1^,^2^,^3^. When working with biological samples, an inherent difficulty of SR-based imaging experiments is associated with the need to position, manipulate and observe the biological sample while keeping it in its natural state. In addition, SR-based scanning techniques typically require that the biological sample remains unchanged during a time frame of minutes to hours, during which 2D/3D raster scans are performed by moving the sample through the X-ray beam. In order to be able to perform such analysis on the micro/nanoscopic level, the immobilization of the sample on the motor stage system and the accurate and precise movements of the sample are real challenges.

When performing X-ray microanalysis of biological organisms close to their natural state, two distinct approaches can be followed, i.e., either by modifying the sample and making it suitable for analysis and/or optimizing the applied analytical methodology. Concerning the sample preparation, it is hard to provide a universally applicable procedure, as the sample treatment steps are primarily determined by the biological specimen itself. Consequently, each biological sample asks for careful optimization and testing of the preparation procedure to minimize artefacts. Moreover, the presence of high amounts of water in most biological samples poses a major challenge due to dehydration and chemical deterioration effects^4^. Many biological sample preparation methods are currently available to remove the water content, however possibly inducing changes in the measured elemental distributions and possibly introducing sample contamination^5^,^6^,^7^. As water has a high surface tension, air-drying of biological specimen should be avoided to prevent cell collapse, aggregation and shrinkage. Other approaches consist of embedding the biological specimen in a medium that later hardens to form a solid matrix^8^,^9^. Note that the employed chemicals should be of low viscosity, preserve the fine structure, retain the chemical identity, be relatively inexpensive and maintain stable in the X-ray beam. For biological organisms a more general approach consists of applying low-temperature specimen preparation, thereby working below the glass transition temperature of water (−137°C). Associated difficulties involve devitrification and subsequent recrystallization events in the course of the sample preparation procedure and during the storage afterwards. In conclusion, ideally the specimen is analysed immediately after completing the sample preparation procedure, which is not straightforward when working at synchrotron environments, as all of the above-listed sample preparation steps ask for a dedicated laboratory, trained personnel and strict health and safety regulations due to the involvement of hazardous chemicals^10^,^11^.

In this paper, we propose a radically new optical tweezers-based micro-XRF methodology that enables two-dimensional (2D) *in vivo* elemental imaging in free-standing biological model organisms and single cells, to be studied in their natural aqueous environment. We thereby eliminate the need for sample preparation steps that may alter the sample and potentially distort the obtained elemental information. Also, the need to mount/fix the cells using potentially interfering sample support materials is eliminated. For the first time, confocal XRF microimaging was combined with optical tweezers (OT) technology for synchrotron radiation based *in vivo/in situ* elemental analysis.

In short, optical tweezers use a focused laser beam to optically manipulate a sample within an aqueous environment, enabling non-contact sample manipulation and positioning. At present, optical tweezers are routinely applied in different fields of science, ranging from physics to life sciences^12^,^13^,^14^,^15^. Applications of this novel *in vivo* OT micro-XRF methodology are expected to enter many research fields, ranging from environmental to life sciences, we tested and optimized this technique while conducting research within the field of environmental toxicology. In particular, we relied on the OT XRF methodology to study the exposure effects of toxic concentrations of transition metals on the microalgae *Scrippsiella trochoidea*. Using this biological model organism, we may gain a better understanding of the complex biotic and abiotic factors that govern the occurrence of this type of harmful algae. To date, the connection between accumulation and the toxic effects of industrial pollution (e.g., heavy metals) on marine microalgae are poorly understood^16^. Despite of this, it is assumed this type of microalgae and their cysts are good indicators of water quality and pollution^17^,^18^.

Here, we report on the results of two subsequent experiments that were conducted at the European Synchrotron Radiation Facility (ESRF, Grenoble, France) ID13 Microfocus beamline to show the potential of the new OT micro-XRF methodology. In a first proof of principle (POP) experiment, we examined the possibilities of OT micro-XRF imaging on *S. trochoidea* microalgae exposed to Ni, Cu and Zn and in a second experiment, the subcellular distribution and toxic effects of Cu-Ni and Cu-Zn binary mixtures were investigated. In addition, the OT XRF methodology was successfully combined with complementary integrated Small Angle X-ray Scattering (SAXS) scanning techniques enabling the visualization of the sample outline.

## Results

This study contains the very first results concerning the new OT XRF methodology that enables the investigation of *S. trochoidea* microalgae under *in vivo* conditions. The samples referred to were optically manipulated and positioned using the OT setup while being measured using SR confocal micro-XRF.

### Microalgae and culturing conditions

While demonstrating the potential of the new OT XRF methodology, we simultaneously aimed to improve our knowledge on the toxic effects of heavy metal pollution on harmful dinoflagellates^19^. To this end, we exposed the marine dinoflagellate *S. trochoidea* to toxic concentrations of Ni, Cu and Zn. This bloom-forming cosmopolitan species was considered a valuable model organism for other toxic dinoflagellates because of its physiological features and its ability to produce cysts^20^,^21^. For the POP experiment, three cultures were exposed to toxic concentrations of transition metals (Zn, Cu and Ni, 100 μg/L in L1 medium www.ccap.ac.uk, 96 h exposure time) while one non-exposed culture served as a reference.

In a second experiment, the mixture toxicity of Cu-Ni (case 1) and Cu-Zn (case 2) on *S. trochoidea* was investigated (96 h exposure time), experimental design in Table 1.

### Compact optical tweezers setup

**Figure 1:**
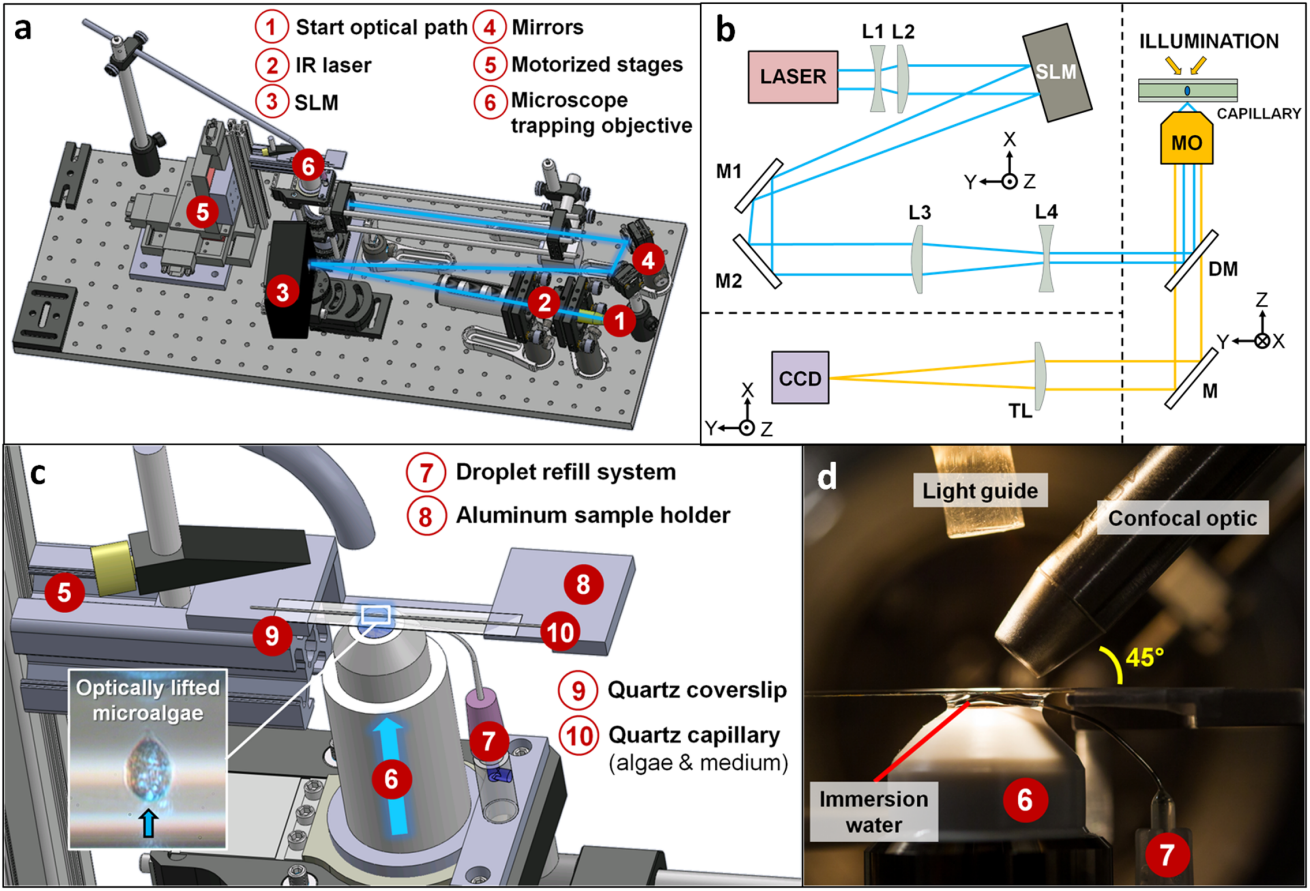
Overview of the compact OT setup. (a) CAD design with an indication of the IR laser path (blue). (b) Block diagram of the optical path in the OT setup. (c) CAD design showing a detail of the sample area. (d) Photograph of the sample environment, confocal optic is positioned under 45°. Note: Technical drawings made in SolidWorks by the main author, drawings of selected components were acquired from THORLABS, Inc. © (http://www.thorlabs.de/).

Three components are essential on an optical tweezers setup: a laser, a microscope objective with high numerical aperture (NA ≥ 1) and an imaging system (Supplementary Video 1). A spatial light modulator (SLM) is also integrated in the compact OT setup to enable the creation of multiple optical traps via phase modulation. This multitude of traps results in a more stable optical manipulation condition as the sample is supported via multiple points in space. In 2011, Santucci *et al.* reported on a dedicated OT setup for SR microdiffraction experiments of soft matter objects in their natural, aqueous environments at the ESRF-ID13 Microfocus beamline^22^. During the past years, we further optimized this compact OT setup to match the spatial requirements for OT-based SR confocal XRF imaging. A CAD design in SolidWorks (Fig. 1a) and block diagram (Fig. 1b) give an overview of the optimized OT setup and the corresponding laser path.

The samples, algae and medium, are contained in a quartz capillary which is taped onto a quartz coverslip and mounted onto an aluminum holder that acts as a stable support (Fig. 1c). It should be noted that all glassware in the vicinity of the trapping objective is of high purity quartz to minimize spectral contributions.

### Confocal XRF imaging and integrated SAXS imaging

The methodological developments required for testing the new OT micro-XRF methodology were carried out at the ESRF-ID13 Microfocus beamline. A transfocator with a set of parabolic Be-refractive lenses^23^ focuses the X-ray beam at an energy of 13 keV and depending on the synchrotron bunch mode, a variable flux and X-ray spot are obtained. During the first proof of principle experiment, a 16 bunch-mode was operational; providing a 2 × 2 μm^2^ primary X-ray beam at a flux of approximately 10^11^ photons/s. For the mixture toxicity experiment, a 4 bunch-mode was in operation, delivering a 2 × 3 μm^2^ focused beam at a flux of approximately 2 × 10^10^ photons/s.

Fluorescent photons were detected using a VORTEX-EM detector tilted to an angle of 45° (with respect to the plane of linear polarization) and perpendicular to the primary X-ray beam. The VORTEX-EM detector was equipped with polycapillary optics (Fig. 1d) thus resulting in a confocal XRF detection geometry with an acceptance of 50–100 μm in order to minimize the fluorescent/scatter signal from the surrounding sample environment.

**Figure 2:**
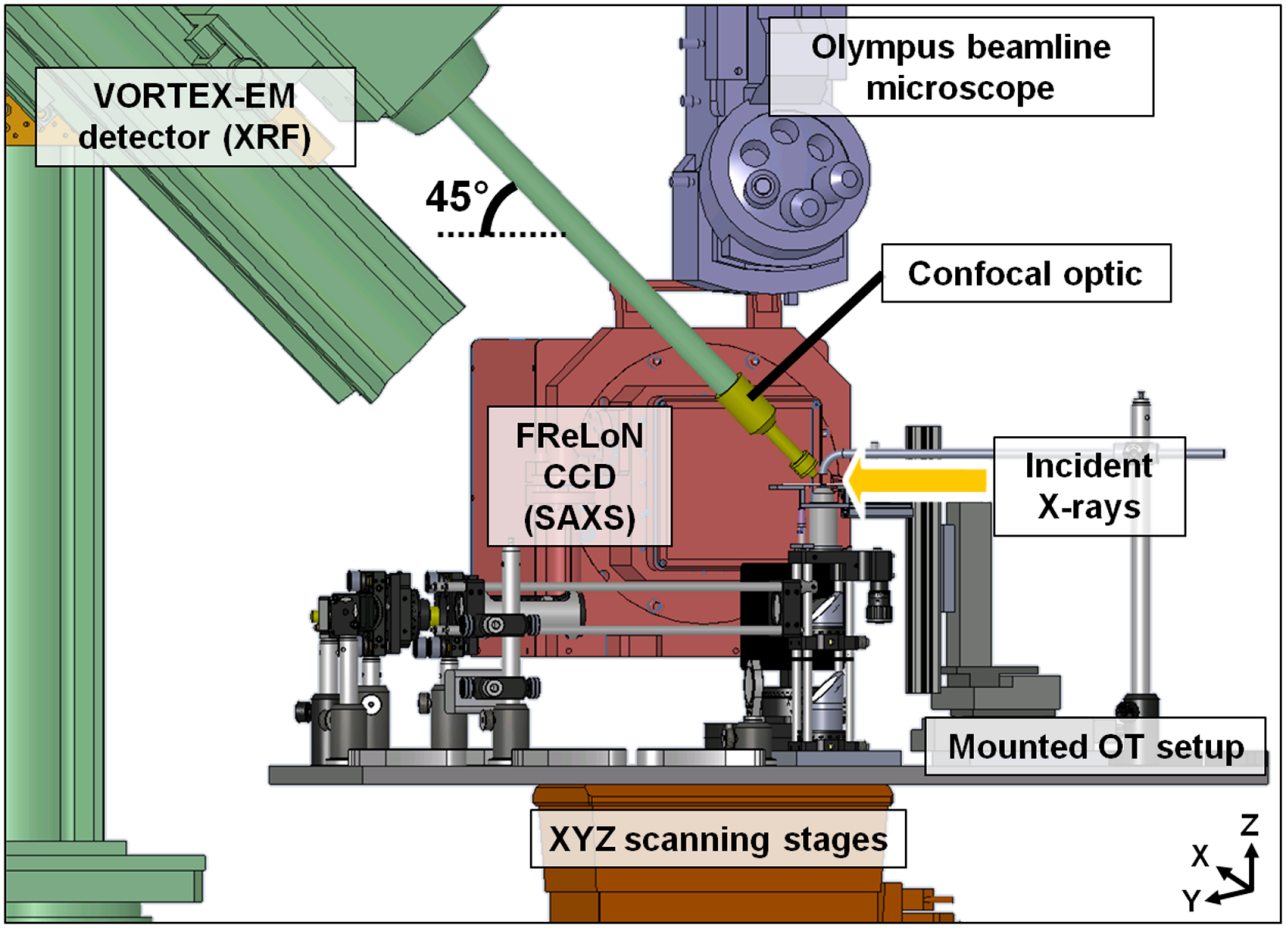
CAD design in SolidWorks showing an overview of the compact OT setup installed at Microfocus beamline ID13. The Olympus beamline microscope is used for aligning the optically manipulated microalgae to the primary X-ray beam. A VORTEX-EM detector collects the fluorescent signal under an angle of 45° (with respect to the plane of linear polarisation), the FReLoN CCD camera collects the SAXS signal downstream (with respect to the primary X-ray beam).

The OT XRF methodology was successfully combined with complementary integrated SAXS imaging that enables to probe the micro/nanoscopic structure and density fluctuations of soft matter objects^24^,^25^,^26^. Within this study the integrated SAXS results perfectly enabled to visualize the microalgae outline which was subsequently used for combined and improved semi-quantitative data processing (Supplementary Fig. S1). The SAXS patterns were recorded by a 16 bit readout FReLoN charge-coupled device (CCD) detector^27^ in transmission geometry using an exposure time of 0.5 s/pattern (Fig. 2).

For X-ray based scanning purposes, a raster scan is performed by displacing the complete OT setup with the beamline YZ stages (Fig. 2) by a fixed amount between acquisitions, thereby forming a grid of measurements on the samples. Optical microscopic images of the samples were obtained either off-line by an Olympus microscope calibrated on-axis to the beam position or on-line by a CCD camera observing the sample through the trapping objective, perpendicular to the primary X-ray beam.

### Proof of principle: OT confocal micro-XRF analysis of *S. trochoidea*

**Figure 3:**
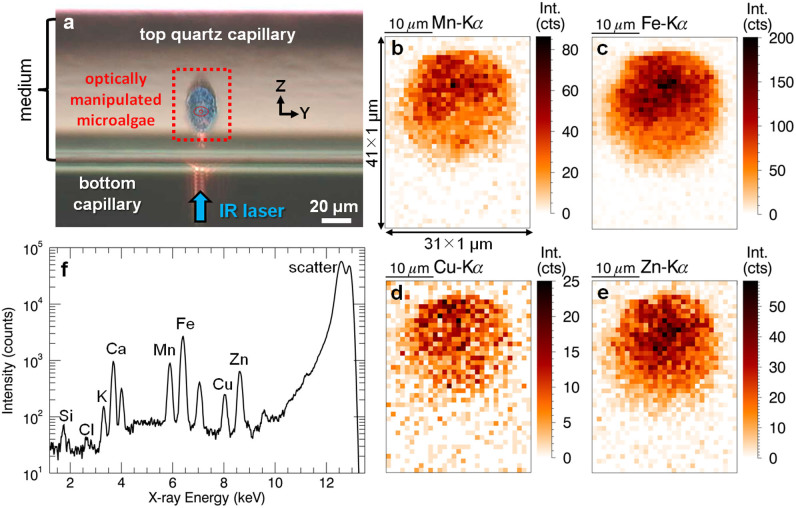
POP experimental results of a scanned Zn-exposed microalgae. (a) Microscopic image of an optically manipulated algae in a quartz capillary, only for demonstration purposes, not corresponding to the actually scanned algae. (b-c-d-e) Mn, Fe, Cu and Zn elemental distributions corresponding to the area scanned with high resolution. (f) Sum spectrum of the scanned area.

Within the POP experiment, four cultures of S. *trochoidea* microalgae were prepared (Zn, Cu and Ni, 100 μg/L, L1 medium, 96 h) and one reference. The microalgae were optically manipulated using a 0.2–0.3 W laser power (one trap or multiple traps are applied depending on the algae size), positioned into the X-ray beam and scanned with SR confocal micro-XRF. The area corresponding to the dotted rectangle on the microscopic image (Fig. 3a) is scanned with high resolution (1 μm step size, 0.1 s/point). Note that on the microscopic image, an optical elongation effect (≈30%) in the vertical scanning direction (Z) is observed due to the cylindrical quartz capillary acting as a concave lens. The total time per scan varied between 5 and 10 min demonstrating the high-throughput potential of the OT micro-XRF methodology.

The elemental distributions show that significant amounts of Mn (Fig. 3b), Fe (Fig. 3c), Cu (Fig. 3d) and Zn (Fig. 3e) were detected within the Zn-exposed algae. These transition metals are essential micronutrients for the growth and metabolism of the microalgae as they fulfill critical roles in photosynthesis and the assimilation of essential macronutrients (e.g., N, P). Of the above-listed micronutrient metals, Fe (Fig. 3c) is needed in the greatest amount to maintain essential metabolic functions in the photosynthetic electron transport, respiration, nitrate assimilation, N_2_-fixation and detoxification of reactive oxygen species. The presence of Mn (Fig. 3b) can be explained by its essential role in photosynthesis and its presence in oxidative stress enzymes. The detected amount of Mn is lower compared to Fe, mainly reflecting its fewer metabolic functions. The Cu-signal is largely explained by the proteins cytochrome oxidase and plastocyanin, both playing a key role in respiratory electron transport while Zn serves in a variety of metabolic functions. Finally, the sum spectrum (Fig. 3f) indicates that significant amounts of Si, K and Ca were present within the scanned area, originating from the quartz capillary (Si), growth medium (Ca, K) en to a lesser extent from the algae^16^,^28^,^29^.

From the OT micro-XRF experiments on the Ni and Cu exposed cultures, similar qualitative elemental distributions were obtained, however no significant Ni signal was measured in the reference samples. Note that Ni is also an essential micronutrient, playing an important role in nitrogen assimilation; therefore naturally present *S. trochoidea,* but in amounts below the XRF limit of detection (LOD).

### Metal mixture toxicity: OT micro-XRF analysis complemented by integrated SAXS

We examined the combination of the new OT micro-XRF methodology with complementary integrated SAXS scanning measurements for sample outline visualization. Moreover, the mixture toxicity on *S. trochoidea* microalgae was investigated in order to retrieve a possible synergistic or antagonistic effect, i.e. the respectively non-linear increase or decrease of the total toxicity when adding a second stressor (e.g., transition metal).

Within two case studies, *S. trochoidea* was subjected to the combined exposure of Cu-Ni (case 1) and Cu-Zn (case 2) using six conditions (1× reference, 2× Cu, 2× Ni/Zn, 1× mix, L1 medium, 96 h). For each exposure condition, three algae were selected and subjected to OT-based X-ray analysis. Similar to the POP experiment, the microalgae were initially lifted using a 0.2–0.3 W laser power and scanned through the X-ray beam while simultaneously collecting the fluorescent signal (perpendicular to primary beam) and scattered signal (downstream) (2 μm step size, 0.5 s/point). The total time per scan varied between 10 and 15 min due to the 4-buch mode of the synchrotron ring.

**Figure 4:**
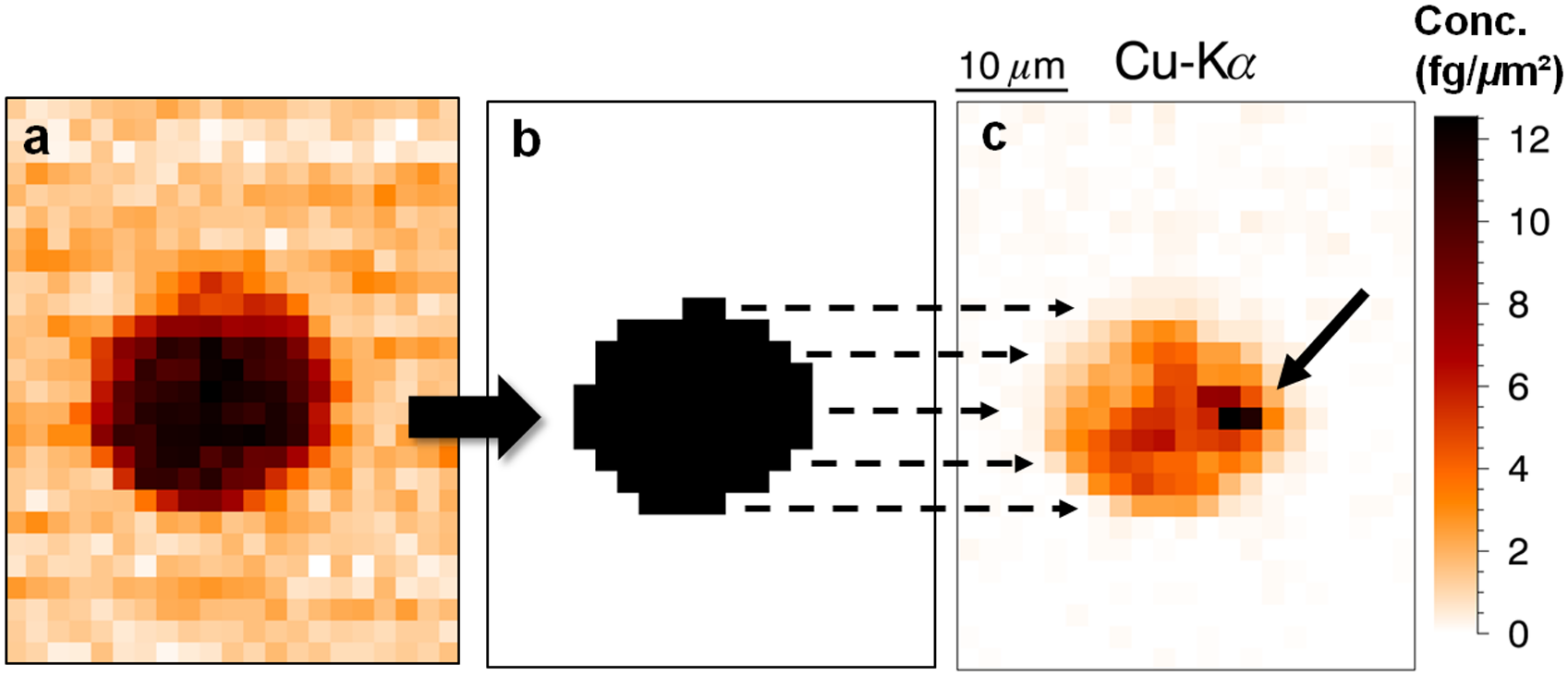
Mixture toxicity experimental results. (a) Integrated SAXS distribution map of a scanned algae. (b) Algae isolated from the integrated SAXS distribution map (after smoothing function and threshold = μ + σ). (c) Cu areal concentration map of a scan on Cu-exposed algae, arrow indicates accumulation centre (case 2, 675 μg/L, values in fg/μm^2^).

The recorded integrated SAXS patterns (Fig. 4a) gave a clear indication of the algae outline and were used as a basis for the segmentation of the elemental maps into algal outline and background regions. Data processing involved isolating the microalgae from the SAXS distribution map (Fig. 4b), followed by a projection onto the XRF elemental maps (Fig. 4c). For each element, the fluorescent signal corresponding to the algae was summed up and normalized by the number of pixels representing the algae, thereby correcting for the biological variability in size. Semi-quantitative attempts were made by using a NIST SRM 1577c (Bovine liver) pressed pellet that was measured for 250 s in confocal geometry. As the standard was measured under ambient air conditions, corrections were applied to the algal data for absorption by the microalgae itself and by the quartz capillary wall. The corresponding areal concentrations in fg/μm^2^ (per pixel) are presented in Table 2.

#### Case 1, Cu-Ni exposure

Within the reference samples, significant amounts of Cu were detected reflecting its essential nature in specific biochemical functions. Ni on the other hand was not detected in the reference samples which is consistent with the POP experiment. As an essential micronutrient, Ni is likely to be present in concentrations below the OT XRF limit of detection. At sublethal concentrations, intracellular concentrations of both Cu (B, E, Table 2) and Ni (C, F, Table 2) increased in a dose-dependent manner. Cu accumulated more readily which could be explained by the formation of chlorophyllin-copper complex (Supplementary Fig. S2). Within the reactive core of Chlorophyll a, the captured Mg^2+^ ion can be interchanged with Cu^2+^, resulting in partial to complete inhibition of photosynthesis. This mechanism is likely to explain the sensitivity of S. *trochoidea* towards the presence of free copper. Moreover, an inhomogeneous distribution of Cu can be seen on the 675 μg/L Cu elemental maps (Fig. 4c, arrow indication). This Cu-rich region may indicate an important organelle (e.g. nucleus, Golgi apparatus etc.^21^), however a more detailed interpretation of this accumulation centre asks for further experimental evidence.

Note that no statistically significant differences in intracellular concentrations of Cu and Ni were found between the single and binary exposure treatments. Nevertheless, the algal cell count data (Supplementary Table S1) clearly suggests that the toxic effect of Cu is greatly enhanced in the presence of Ni. Crucially, the algal areal concentrations prove that both transition metals were present at intracellular levels doses similar to those of the single metal exposures, thus providing strong support for this hitherto unknown synergistic toxic effect of Cu and Ni.

#### Case 2, Cu-Zn exposure

During case 2, the repeatability of our novel method was demonstrated as the internal Cu areal concentrations, under both the reference as the exposure conditions, were found to be very similar to case 1. Zn however, was not found to accumulate significantly relative to the control treatment, and considering the error bars the tested concentrations (I, L, Table 2) are probably still within the tolerable concentration range. Nevertheless, Zn was also found to increase the toxicity of copper, albeit to a lesser extent than Ni (Supplementary Table 1).

**Figure 5:**
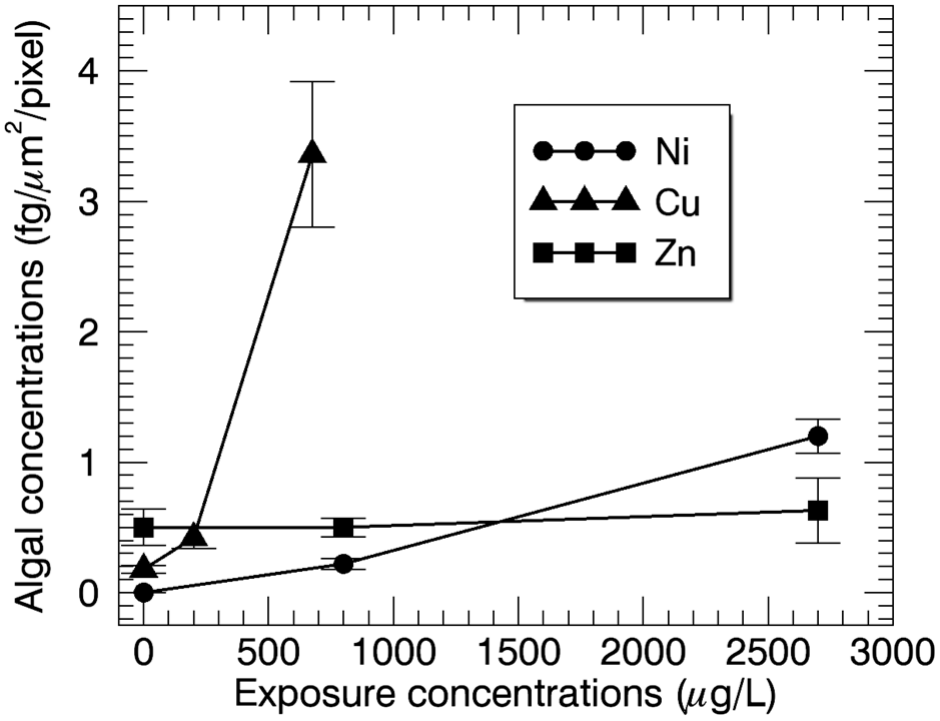
Tendency towards the bioaccumulation of Ni (case 1, dot), Cu (case 2, triangle) and Zn (case 2, square) in *S. trochoidea* microalgae, error bars show s.d.

Overall, it can be concluded from the binary mixture experimental results (Table 2) that large differences exist in algal sensitivity towards the bioaccumulation of metals. A graphical representation (Fig. 5) shows the following trend in accumulation behaviour: Cu ≫ Ni > Zn.

### X-ray sample damage

Due to the microscopic size of the primary X-ray beam and its high flux (10^10^–10^11^ photons/s), X-ray radiation damage of the microalgae cannot be fully eliminated. Radiation damage is caused by absorbed X-ray photons depositing energy directly within the sample, mainly causing inner orbital electrons to be ejected due to the photoelectric effect. As a result of the predominant K-shell ionization of low atomic number elements, a large number of electrons having energies close to the incident photon energy are produced, as well as Auger electrons characterized by lower energies. Many of the produced electrons will escape from the irradiated sample volume, while a substantial amount of these negatively charged particles will cause secondary ionization events giving rise to a net positive charge. All these effects jointly result in the photoelectric effect being the primary source of sample damage^30^,^31^.

For this study, an X-ray absorbed dose of approximately 10^6^ Gy on a single scanning point was used, assuming a mass density close to that of water, a 2 μm scanning step size and an incoming intensity of 2 × 10^10^ photons/s^32^,^33^. As a result of the impinging X-rays, basic chemical phenomena such as the photoelectric effect and the subsequent breaking of chemical bonds, take place within hundreds of femtoseconds after the start-up of a scan. Overall effects of the consequent X-ray sample damage include a change of the optical properties, charging effects and detectable elemental mass losses; altogether posing strong constraints on the dose and scanning speeds that can be used while limiting the achievable spatial resolution on delicate biological samples^34^.

Regarding the optical stability, we observed X-ray induced optical changes which may in turn influence the positioning stability in the vertical scanning direction, potentially resulting in incomplete/distorted scans on the investigated cells. To improve vertical stability during the scans, the microalgae is trapped and moved upwards, in contact with the upper capillary wall, providing mechanical support and thereby preventing a vertical sample movement. This position also results in the reduction of the thickness of the aqueous medium layer between the investigated cell and the detector, minimizing absorption effects of the detected fluorescent signals.

Furthermore, radiation damage related phenomena induce (limited) XRF signal losses during a progressing scan. These XRF signal losses are element dependent, having a maximum loss of 40% for the most sensitive elements under non-optimized scanning conditions. Consecutive scans on a single microalgae showed that certain elements preferentially escape from the organism (e.g., Mn) while others tend to be concentrated (e.g., Cu). We can therefore conclude that scanning X-ray methodologies that require multiple passes over the same biological organism, such as X-ray fluorescence computed tomography (XRF-CT), are difficult to realize with the current OT XRF methodology. However, less delicate microsamples with appropriate optical and X-ray damage resistant properties will most likely be suitable for XRF-CT imaging using non-contact sample manipulation by optical tweezers. Examples include solid catalyst particles^35^, particles for water treatment technology^36^ and metallic host particles materials used in battery technology^37^.

In case of single pass scanning approaches, such as the measurements described in this paper, the above mentioned X-ray beam induced sample damage/instabilities can be significantly reduced by further optimizing the applied detection and scanning speeds. In particular the detector load was a critical parameter in our case due to the substantial contribution of the scattered signal to the XRF spectra, partially caused by the 45° inclination angle of the detector. However, with the advances in detector technology and electronics, it is expected that detector throughput capabilities will substantially improve during the next few years. In combination with optimized confocal polycapillary optics having a larger solid angle of acceptance (approximately a factor 10 compared to the currently employed optics), we expect that the overall detection efficiency/throughput can be increased by a factor 10–100 compared to the current conditions. This will enable us to move towards ultrafast scans in the (sub-)millisecond range per point which will result in a significantly reduced influence of X-ray sample damage.

## Discussion

As stated, two distinct approaches can be followed for performing X-ray microanalysis of biological organisms close to the natural state, we focused on optimizing the applied analytical instrumentation by introducing the novel OT XRF methodology. We thereby step away from the generally accepted trend of applying time-consuming, often invasive and error-prone sample preparation steps prior to analysis.

The novel high-throughput OT micro-XRF methodology is very well suited for the *in vivo* elemental micro-XRF analysis of biological organisms and single cells in their natural, aqueous environment. In a first proof of principle experiment, we explored the potential of OT micro-XRF imaging at Microfocus beamline ID13 on the marine microalgae *S. trochoidea* microalgae, which we exposed to the transition metals Ni, Cu and Zn. For our second experiment, the OT XRF methodology was successfully combined with complementary integrated SAXS imaging to visualize the sample outline, simultaneously. Beyond these technical improvements, we demonstrated the practical applicability and repeatability through an ecotoxicological case study. More specifically, the OT micro-XRF method was used to study the effect of binary mixtures of Cu-Ni and Cu-Zn on *S. trochoidea* which led to the discovery of previously unknown synergistic toxicity effects. Crucially, this unique methodology allowed us to semi-quantitatively determine rather large differences (Cu ≫ Ni > Zn) in the *in vivo* areal concentrations of accumulated metals in single cells, measured in 2D projection mode.

In future experiments, the possibilities of direct sample positioning and scanning using the SLM will be explored, ultimately making the complex, high resolution motorized stages obsolete. Moreover, the sample can be slowly rotated by the SLM chip, resulting in the possibility for tomographic imaging on microscopic (non-biologic) samples with appropriate geometrical/compositional properties and X-ray damage resistance. Moreover, further technical improvements can be obtained by combining the OT XRF methodology with other SR-based analytical techniques such as phase-contrast imaging, ptychography, (time-resolved) SAXS or dynamic SAXS. Furthermore, the current sampling procedure can be optimized by introducing a pumping system enabling online acute exposure OT XRF studies on optically mounted biological specimen. In addition, the OT setup can be further miniaturized permitting it to be installed and tested at higher resolution beamlines. A smaller and lighter OT setup would also enable its integration in X-ray laboratory equipment to perform in-house OT XRF analyses, despite of the lower flux and larger X-ray focal spot.

In general, for future OT XRF experiments we propose ultra fast scans on a variety of microscopic samples with a wide range of applications in all disciplines where *in situ*, highly sensitive multi-element analysis are of relevance on the high spatial resolution level.

## Methods

### Optical manipulation of biological model organisms

Optical manipulation is achieved when a highly focused laser beam is directed onto a microscopic dielectric object and arises from the refractive index mismatch of the object with its environment (e.g., water). Depending on the sample size and therefore the active optical forces (in the order of piconewtons), optical manipulation phenomena can be distinguished into two regimes: optical trapping^38^ and optical levitation^39^ (Supplementary Fig. S3 and Video 2).

During our preliminary studies, a large set of biological model organisms was tested for their suitability towards optical manipulation. Microalgae are a class of model organisms that generally comply well with the size and compositional requirements for optical manipulation experiments. For example Nishimura *et al.* showed that the microalgae *Chlamydomonas reinhardtii* (fresh water algae, 10 μm) can be optically trapped for extended periods of time (i.e., several hours) to investigate non-Mendelian inheritance of organelle genes^40^. In addition we found that *Pseudokirchneriella subcapitata* microalgae (Korshikov, fresh water algae, 6 μm)^41^ are sensitive towards optical trapping as well. However, a balance should be found between the sample size and the amount of mass available for SR confocal micro-XRF analysis. Therefore several larger microalgae were tested and the marine microalgae S. *trochoidea* (30 μm)^21^ matched well with the size range for both optical manipulation and micro-XRF imaging. Due to its slightly larger size, the optical levitation phenomenon becomes valid when directing a focused laser beam onto *S. trochoidea*.

### Sample preparation

In order to lower the background signal originating from the highly concentrated exposure medium, the algal cultures were shortly washed in a HEPES buffer (1 L deionized water, 32 g NaCl, 3 mM HEPES, pH 8). Furthermore, the sample preparation is kept to a minimum and only involves transferring the microalgae and medium to cylindrical capillaries by means of capillary forces. The sample holders are cylindrical quartz capillaries (QGCT 0.1, Capillary Tube Supplies Ltd, UK, 100 μm diameter, 10 μm wall thickness) which are open at both ends. Once filled, putty is used to seal the capillaries to overcome evaporation of the medium. Afterwards the capillaries are cleaned using cotton swabs immersed in high purity ethanol (Ethanol, ROTISOLV HPLC Gradient Grade, Carl Roth) and positioned onto a thin UV fused silica Spectrosil 2000 quartz coverslip (UQG Optics Ltd., 40 mm × 5 mm × 0.10 mm thickness). The quartz capillaries are taped onto the coverslip using Scotch tape and finally mounted onto an aluminum holder that acts as a stable support (Fig. 1c). It should be noted that the sample preparation is executed only shortly before the sample is transferred to the OT setup and X-ray scans are performed.

### Compact OT setup

All optical components are mounted on a THORLABS optical breadboard (650 mm × 250 mm × 12.7 mm) that can be fixed to the Micos UPL-160 elevation stage of Microfocus beamline ID13. The laser fiber represents the start of the optical path and should therefore be stably fixed and properly aligned. A YLM-5-LP-SC ytterbium fiber laser source (IPG Photonics, USA) delivers a collimated, linearly polarized cw TEM_00_ beam at the single mode 1070 nm wavelength which induces negligible biological damage^42^. A second red laser (≈650 nm wavelength) is available in the laser unit for initial alignment purposes. A beam expander is positioned after the laser fiber to maximally illuminate the SLM chip which is positioned in upright geometry (Supplementary Fig. S4). The SLM is computer-controlled and uses holograms to modulate the phase of the initial laser beam. The required holograms are generated via an in-house written IDL-program (Interactive Data Language, Exelis Visual Information Solutions, Boulder, Colorado, www.exelisvis.com) based on the iterative Gerchberg-Saxton algorithm^43^. The laser beam is further guided towards the Olympus 100× LUMPlanFl water immersion microscope objective (MO) after passing through a set of mirrors and lenses. Prior to entering the trapping objective a dichroic mirror (DM) is present that reflects photons at the wavelength of 1070 nm, but is transparent towards visible light.

The back aperture of the water immersion objective is overfilled and focuses the IR laser beam to a diffraction limited spot in which objects can be optically manipulated. The trapping objective has a high numerical aperture (NA = 1) and a working distance of ≈1 mm, a water droplet on top of the objective is needed in order to focus the IR laser beam properly. To compensate for the water evaporation, a droplet refill system was installed consisting of a bend needle (Sterican), an in-house made adapter piece and an ALADDIN2-220 (World Precision Instruments) syringe pump, providing a constant flow of 35 μL/h of Milli-Q water. Using this flow rate the water droplet volume can be maintained at a constant level for several hours. For sample observation, a CCD camera from Watec (WAT-221S) is installed on the optical breadboard that receives visible light photons from the light guide positioned on top of the MO. The visible light photons travel through the trapping objective in the opposite way of the IR laser photons. In front of the CCD camera, a tube lens (TL) couples the infinity-corrected microscope objective to the CCD camera.

### Sample manipulation

Sample manipulation is subdivided into: motorized manipulation and optical manipulation. Motorized movements of the aluminum sample support are performed by the XYZ motor stages (Micos MT-60, Fig. 1a) that are integrated on the optical breadboard. By moving these motor stages, the capillary is positioned on top of the trapping objective into the laser focal spot (accompanied by sample observation via the CCD camera).

Optical manipulation is performed by adapting the applied laser power. In the optical levitation regime the sample moves upwards with increasing laser power; a downward movement is observed with decreasing laser power as a consequence of the decreasing scattering force and constant gravitational forces. In addition, optical manipulation can be performed using the SLM and a set of holograms, resulting in a shift of the laser focal spot (in X, Y and Z relative to the primary X-ray beam). Due to the shifted laser beam, the sample moves as if the complete OT setup would be translated. The SLM (and a set of holograms) can therefore mimic the beamline stages movements ultimately making the complex nanoscopic motor stages obsolete. Moreover, the sample can be slowly rotated by rotating the hologram that is sent to the SLM chip, resulting in the possibility for OT XRF tomographic imaging on microscopic (non-biologic) samples with appropriate geometrical/compositional properties and X-ray damage resistance.

### Optical stability

Since the OT setup was combined with XRF and complementary integrated SAXS imaging on the micro level, a stability study of a microalgae in the optical trap is crucial. While the OT setup was installed at Microfocus beamline ID13, the on-axis Olympus beamline microscope (Fig. 2) collected images of an optically lifted sample every 0.1 s (for 15 min, typical scan duration). Data processing of the image collection is performed using in-house software developed in the IDL programming software. The JPEG-files are read in and four regions of interest (ROI) are selected from which four points of interest (POI) are extracted (Supplementary Fig. S5): two positions on the microalgae (POI 1 & 2), a position of the lower capillary wall (POI 3) and the position of the laser beam (POI 4). During a typical scan of 15 min, an average sample deviation of 0.23 μm is observed in the horizontal scanning direction (Y) and 0.84 μm in the vertical scanning direction (Z). Taking the average size of a *S. trochoidea* microalgae into account (i.e., 30 μm), it can be concluded that sufficient submicron optical stability is available in both scanning directions for micro-XRF and integrated SAXS imaging.

### Micro-XRF confocal imaging

Due to spectral contributions coming from the optical components (microscope objective, coverslip, aluminum sample support etc.) conventional micro-XRF is not feasible. Therefore a polycapillary half-lens (XOS, X-ray Optical Systems Inc., Albany, USA) was positioned in front of the energy-dispersive VORTEX-EM detector (50 mm^2^ active area, 350 μm crystal thickness, 2 μs peaking time, HITACHI, USA), resulting in confocal XRF mode^1^. The applied confocal XRF methodology is crucial to reduce/eliminate the interfering fluorescence/scatter signal from the surrounding sample environment, since the detection volume is confined to the intersection of the exciting beam and the energy-dependent acceptance of the polycapillary. Minimum detection limits are sub-ppm and sensitivities are comparable with regular scanning XRF^1^. For the POP experiment, an XOS confocal optic with a 3.5 mm working distance (WD) was installed and aligned to a ≈50 μm^3^ detection volume (Fig. 1d and Supplementary Fig. S6). For the mixture toxicity study, an XOS confocal optic with a 10 mm WD was used and aligned to a ≈100 μm^3^ detection volume. Due to the short working distances of the confocal optics, the VORTEX detector is tilted to an angle of 45° (with respect to the plane of linear polarization) causing minimal absorption of the fluorescent signal by the quartz capillary. The 10 mm WD confocal optic provides more spatial freedom for sample exchange and simplifies the confocal alignment due to the larger confocal volume.

## Author Contributions

E.V. developed and optimized the OT XRF methodology for several biological model organisms, she conducted and managed the SR XRF experiments at ESRF ID13 and wrote the manuscript. T.B. and F.B. optimized the OT setup for SR XRF experiments and performed the OT alignment during the initial experiments at ESRF ID13. J.G. contributed in the technical SR XRF developments, both J.G. and S.B. assisted during the scanning experiments at ESRF ID13. M.D.R., D.D., M.V. and C.J. provided the microalgae samples, developed the biological experimental design and exposed the cell cultures to transition metals. I.V.N. assisted in the gelatine fixation procedure. M.B. provided the loan of the OT setup and allowed its optimization, he performed the initial X-ray beam optimization and proposed the combination with integrated SAXS imaging. L.V. proposed the concept of confocal XRF experiments combined with the OT setup and assisted during the initial experiments at ESRF ID13. All authors reviewed the manuscript.

## Supplementary Material

Supplementary Information Supplemenatry information

Supplementary Information Supplementary Video 1

Supplementary Information Supplementary Video 2

## Figures and Tables

**Table 1:** Experimental design of the metal mixture toxicity study on *S. trochoidea* microalgae (characters indicate tested concentrations). Case 1: Cu and Ni, case 2: Cu and Zn

		Case 1: Ni			Case 2: Zn		
		0 μg/L	800 μg/L	2700 μg/L	0 μg/L	800 μg/L	2700 μg/L
Cu	0 μg/L	A	C	F	G	I	L
	200 μg/L	B	D		H	J	
	675 μg/L	E			K		

**Table 2:** Algal areal concentrations of the mixture toxicity study, values expressed in fg/μm^2^, error bars show s.d.

Case 1: Cu-Ni				Case 2: Cu-Zn			
Binary mixture concentrations		Cu (fg/μm^2^)	Ni (fg/μm^2^)	Binary mixture concentrations		Cu (fg/μm^2^)	Zn (fg/μm^2^)
A	Control	0.20 ± 0.02	<LOD	G	Control	0.18 ± 0.03	0.50 ± 0.14
B	200 μg/L Cu	0.39 ± 0.08	<LOD	H	200 μg/L Cu	0.42 ± 0.08	0.47 ± 0.29
C	800 μg/L Ni	0.13 ± 0.01	0.22 ± 0.04	I	800 μg/L Zn	0.20 ± 0.08	0.50 ± 0.07
D	B + C	0.31 ± 0.04	0.10 ± 0.09	J	H + I	0.31 ± 0.03	0.38 ± 0.05
E	675 μg/L Cu	3.04 ± 0.58	<LOD	K	675 μg/L Cu	3.36 ± 0.56	0.43 ± 0.10
F	2700 μg/L Ni	0.28 ± 0.08	1.20 ± 0.13	L	2700 μg/L Zn	0.24 ± 0.06	0.63 ± 0.25
