# Supplementary material for: In vivo X-ray elemental imaging of single cell model organisms manipulated by laser-based optical tweezers
Source: Sci Rep. 2015 Mar 12;5:9049. doi: 10.1038/srep09049 (PMC4356986; doi:10.1038/srep09049)
Supplement: Supplementary Information — Supplemenatry information [file srep09049-s1.pdf]

Supplementary information: ***In vivo*** **X-ray elemental imaging of single cell model organisms manipulated by laser-based optical tweezers**

Eva VERGUCHT, Toon BRANS, Filip BEUNIS, Jan GARREVOET, Maarten DE RIJCKE, Stephen BAUTERS, David DERUYTTER, Michiel VANDEGEHUCHTE, Ine VAN NIEUWENHOVE, Colin JANSSEN, Manfred BURGHAMMER and Laszlo VINCZE

Video 1 - Compact OT setup

Overview of the compact OT setup in SolidWorks with an indication of the most important optomechanical components.

Data processing

Spectral deconvolution of the individual XRF spectra was performed by the nonlinear least-squares fitting software AXIL [1]. The batch processing of the individual XRF spectra was performed using the MicroXRF2 software package written in IDL. The flowchart (Supplementary Fig. S1) gives an overview of the steps applied for the combined XRF and integrated SAXS data treatment.

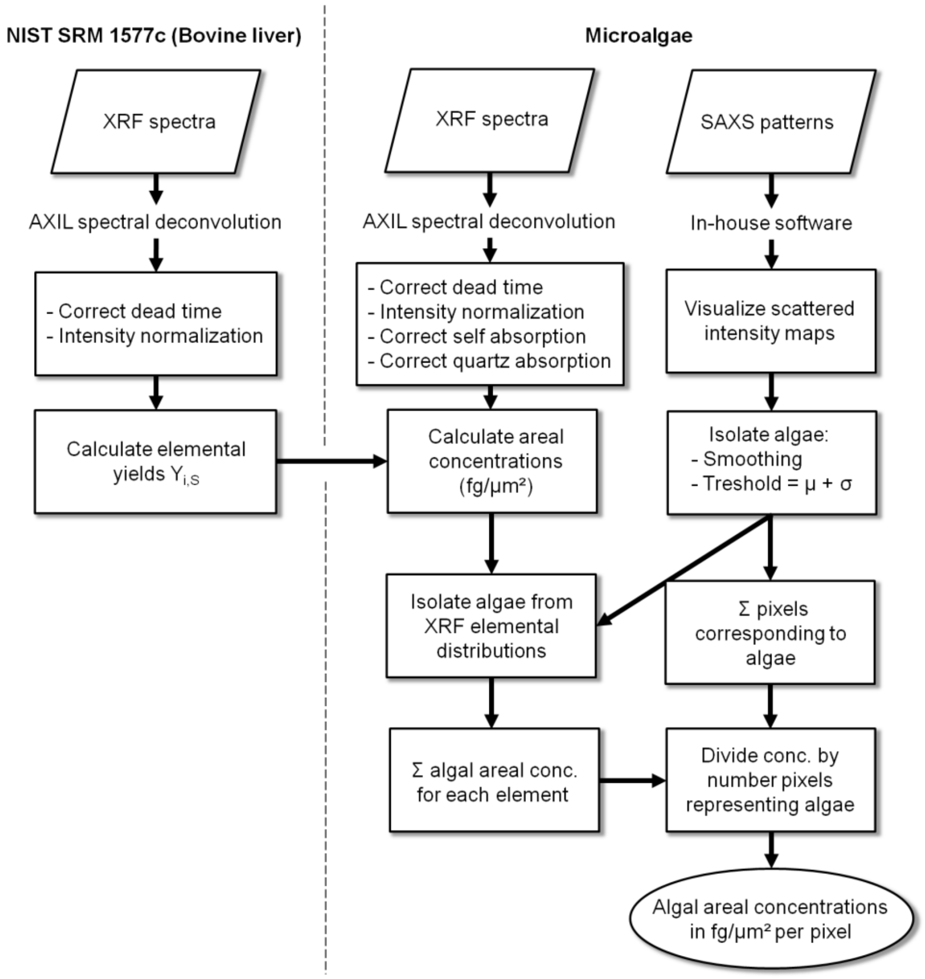

Figure S1: Data processing flowchart.

Chlorophyllin Copper Complex

Chlorophyll a has a reactive core which contains a Mg^{2+} ion captured in a N-ring. The Mg^{2+} ion can be interchanged with Cu^{2+}, resulting in partial to complete inhibition of photosynthesis processes (Supplementary Fig. S2). This mechanism is very likely to explain the high sensitivity of S. *trochoidea* microalgae towards the accumulation of free copper.

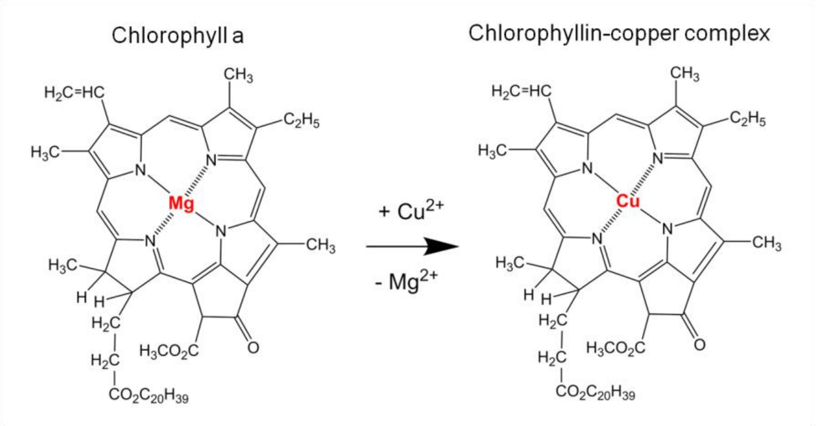

Figure S2: Formation of the chlorophyllin copper complex by exchanging the central Mg^{2+} ion with a Cu^{2+} ion (http://www.nullo.com/chlorophyllin.html).

Algal cell count data

The experimental design of the mixture toxicity study included 24 treatments. Since certain binary mixture concentrations caused cell death throughout the 96 h of exposure, only a limited number of combinations were measured using the new OT XRF methodology. These six treatments are highlighted in Table S1. Initial cell densities (prior to exposure) of the cultures were on average 0.14 cells/µL and 0.29 cells/µL for case 1 and case 2 respectively. After 96h of exposure, all cultures were counted five times. Note that a clear synergistic effect can be observed from the algal cell count data, especially for the Cu-Ni binary mixtures (Supplementary Table S1).

Sample number	Binary mixture concentrations		Cell density (cells/µL) Case 1: Cu-Ni	Cell density (cells/µL) Case 2: Cu-Zn
	Cu (µg/L)	Ni or Zn (µg/L)		
1	0	0	0.75 ± 0.16	1.74 ± 0.37
2	200	0	0.71 ± 0.12	1.23 ± 0.12
3	300	0	0.51 ± 0.13	1.31 ± 0.25
4	450	0	0.79 ± 0.09	1.77 ± 0.21
5	675	0	0.71 ± 0.17	1.71 ± 0.12
6	1012.5	0	0	0
7	0	800	0.59 ± 0.32	0.79 ± 0.20
8	0	1200	0.83 ± 0.11	1.56 ± 0.30
9	0	1800	0.65 ± 0.14	1.54 ± 0.18
10	0	2700	0.36 ± 0.24	1.32 ± 0.32
11	0	4050	0.73 ± 0.10	1.54 ± 0.41
12	200	1200	0	1.29 ± 0.39

13	300	1200	0	1.12 ± 0.08
14	450	1200	0	2.34 ± 0.34
15	675	1200	0	0
16	1012.5	1200	0	0
17	200	2700	0	2.11 ± 0.48
18	300	2700	0	1.89 ± 0.29
19	450	2700	0	0.31 ± 0.08
20	675	2700	0	0
21	1012.5	2700	0	0
22	200	800	0.06 ± 0.04	2.00 ± 0.29
23	450	2700	0	1.92 ± 0.37
24	1012.5	4050	0	0

Table S1: Algal cell count data of the mixture toxicity study.

Optical manipulation

A stable optical (Supplementary Fig. S3) trap is obtained when both the arising scattering force and the gradient force achieve an equilibrium condition. The scattering force arises from the reflection of photons at the sample surface and tends to push the object along the direction of the incoming laser beam. For dielectric particles, also part of the incoming laser light is refracted and thereby resulting in a gradient force in the direction of the spatial light gradient, towards the focus of the laser beam [2]. Below a certain size limit (several Angström up to 10 micrometer) the optical trapping phenomena is valid, to the extent that single atoms were already successfully optically trapped (awarded with Nobel Prize in Physics 1997) [3].

A stable optically levitated sample is obtained when the scattering force is adequate to overcome the gravitational forces. Optical levitation takes therefore place when a highly focused laser beam is directed on a slightly larger sample (10 µm - tens of micrometers).

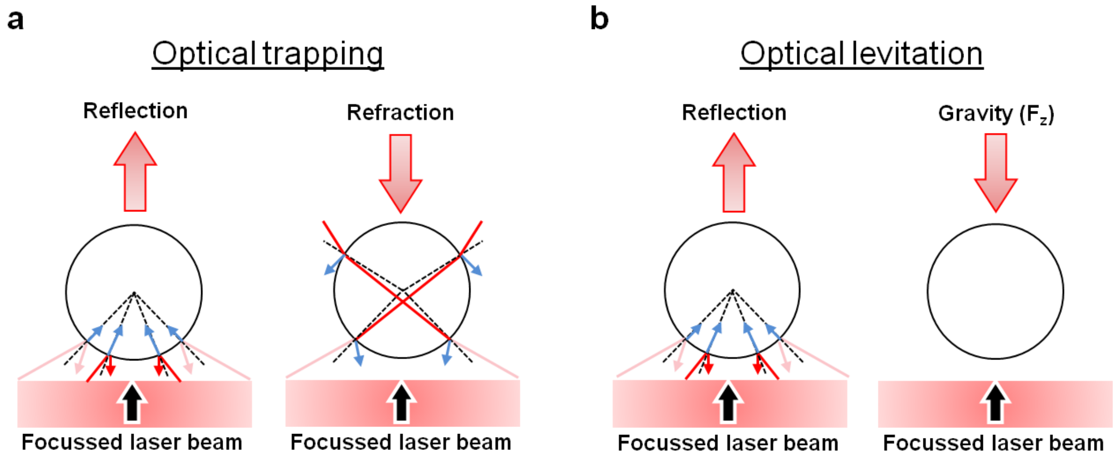

Figure S3: Optical manipulation phenomena. (a) Optical trapping combines reflection and refraction of incoming IR photons [4]. (b) Optical levitation combines reflection of IR photons and gravitational forces for manipulating a slightly larger microscopic object.

Video 2 - Optical trapping and levitation

Demonstration of optical trapping and optical levitation.

- Example 1: optical trapping of a silica microsphere (3.5 µm diameter, Bangs Laboratories, Inc.), translation Y and Z.
- Example 2: optical trapping of two *C. reinhardtii* microalgae (10 µm diameter), rotation of the hologram on the SLM chip.
- Example 3: optical levitation of a *S. trochoidea* microalgae (35 µm width), translation Y.
- Example 4: optical levitation of a *S. trochoidea* microalgae, increasing and decreasing applied laser power.

Spatial Light Modulator (SLM)

The SLM (LCOS-SLM X10468-03 liquid-crystal-on-silicon phase-only SLM, Hamamatsu Photonics, Japan) enables the creation of multiple optical traps via phase modulation, which contributes to a more stable optical manipulation condition as the sample is supported via multiple points in space. The incoming linearly polarized beam is aligned parallel to the orientation of the liquid crystals (upright geometry of the SLM chip, Supplementary Fig. S4).

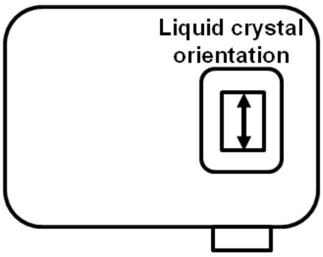

Figure S4: Schematic overview of the SLM orientation and liquid crystal orientation.

Optical stability

Since the combination of OT-based sample manipulation with XRF and complementary integrated SAXS analysis is performed on the microscopic level, a stability study of a microalgae in the optical trap is pivotal. During a typical scan of 15 min (Supplementary Fig. S5), an average sample deviation of 0.23 µm is obtained in the horizontal scanning direction (Y) and 0.84 µm in the vertical scanning direction (Z).

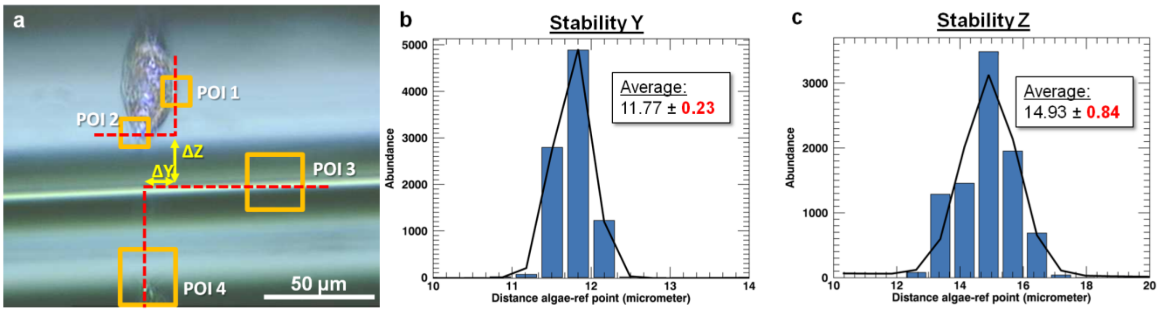

Figure S5: Optical stability study. (a) Microscopic image of an optically levitated algae with an indication of 4 POIs. (b) Histogram for the stability in Y (horizontal scanning direction). (c) Histogram for the stability in Z (vertical scanning direction).

Micro-XRF confocal alignment

For confocal alignment purposes NIST SRM 2066 glass microspheres (size range: 1-40 µm) were used because of their high Fe content (11%). As the glass microspheres cannot be optically manipulated, they were therefore fixed in a gelatin polymer matrix. In brief, the microspheres were added to a home-synthesized methacrylamide modified gelatin monomer solution (Supplementary Fig. S6, gel-MOD, 10 w/v% polymer concentration, 65-70% degree of substitution determined via ^{1}H NMR in D_2O at 40°C) dissolved at 37°C together with 2 mol% Irgacure 2959 initiator. The resulting solution was transferred into a quartz capillary by means of capillary forces and covalently cross-linked via UV initiation (UV-A 250-450 nm, 10 mW/cm^2, 30 min irradiation). Finally, the quartz capillary is taped onto a quartz coverslip and mounted onto an aluminum holder, a similar arrangement as for the microalgae.

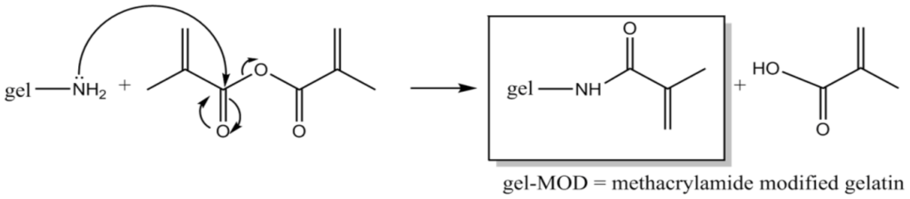

Figure S6: Reaction mechanism for synthesizing methacrylamide modified gelatin (gel-MOD).

References

1. Vekemans, B., Janssens, K., Vincze, L., Adams, F. & Van Espen, P. Analysis of x-ray spectra by iterative least squares (axil) : new developments. *X-ray Spectrom.* **23**, 278-285 (1994).
2. Neuman, K.C. & Block, S.M., Optical trapping. *Rev. Sci. Instrum.* **75**, 2787-2809 (2004).
3. Chu, S., Bjorkholm, J.E., Ashkin, A. & Cable, A., Experimental observation of optically trapped atoms. *Phys. Rev. Lett.* **57**, 314-317 (1986).
4. Santucci, S.C. *et al.*, Optical tweezers for synchrotron radiation probing of trapped biological and soft matter objects in aqueous environments. *Anal. Chem.* **83**, 4863-4870 (2011).
